# Safety and Quality of Generic Drugs: A Never Ending Debate Fostered by Economic Interests?

**DOI:** 10.1007/s40258-015-0156-7

**Published:** 2015-06-20

**Authors:** Livio Garattini, Katelijne van de Vooren

**Affiliations:** CESAV, Center for Health Economics, IRCCS Institute for Pharmacological Research ‘Mario Negri’, Via Camozzi 3, 24020 Ranica, Italy

A generic medicine is a pharmaceutical product intended to be interchangeable with the originator, manufactured without a licence from the innovating company and marketed after expiry of a patent or other exclusivity rights [1]. In principle, a generic medicine should be marketed without a commercial brand name, under the International Non-proprietary Name (INN). However, the definition of generic medicine is not always precise, as it is still more a commercial than a legislative concept in many countries.

Copies of branded drugs have been marketed for decades in many countries; however, laws in the last century such as the Hatch-Waxman Act in the US, have made it much easier and cheaper to bring a new generic drug to market without undermining its quality, safety and efficacy. In Europe, an abbreviated procedure—the abridged application defined by directive 87/21/EEC—is permitted under certain circumstances, therefore generic manufacturers merely have to prove that their drugs have the same active ingredients and perform in the same way as the branded drugs.

The first article [2] of this supplement confirms that the regulatory definition of generics is still inconsistent and piecemeal throughout the world. To warrant the full inter-changeability of generics with originators, their definition should include the requirements for bioequivalence. Unfortunately, important differences in the terms used for generic drugs, such as similars, copies, branded generic products, etc., are still found among countries worldwide. These differences and inconsistencies can challenge the trust of local people and must be addressed and recognised, particularly in developing countries. The second article [3], focused on the Chinese market (one of the most important in the world), shows a very important example of the problems raised by the lack of a clear cut definition of generics in the biggest developing country. It describes a confusing array of drug categories and a lack of quality assurance for generics that could easily be avoided by copying the regulation of developed countries.

A crucial problem for generics is the guarantee on safety and quality of products marketed. Although evidence of bio-equivalence is important, national health authorities should set up mechanisms for checking manufacturing practice by (local and foreign) providers too. However, safety and quality problems should be less of a threat than in the past, at least in developed countries where the few manufacturers (including the “sister companies” of some “big pharma”) in the generics market are definitely interested in avoiding risky practices that could dramatically undermine their image [1]. A peculiar issue brought up in many comments is the distrust of generics due to the high potential for counterfeits in developing countries. However, this argument hardly matters for generics specifically, since counterfeiters are much more likely to go after the higher priced, branded drugs that would offer revenues far exceeding those made by forging much cheaper generics.

Once these basic issues have been addressed, the impact of generic substitution on health and economic outcomes, analysed in the third article [4], would be negligible and its debate pointless. Once it is guaranteed that both originators and generics use an approved and identical active ingredient with a similar bioavailability, the outcomes should be identical and therefore there is no reason to think that generics could end up costing more than their branded equivalent products.

Nevertheless, despite representing over half of the total volume of pharmaceutical products used worldwide [5], scepticism about generics is still widespread, often inspired by adverse economic interests. In general, most pharmaceutical companies have an obvious interest in discrediting generics and undermining their credibility among physicians [6], who in turn are reluctant to favour the widespread prescription of generics without any financial incentive by health authorities. In principle, community pharmacists are more open to using generics. Their interest depends on whether commercial discounts offered by generics manufacturers can compensate their lower margins (compared to originators) and the extra time needed to inform patients.

The last article [7] shows that the attitude of physicians and pharmacists towards generics still varies a lot worldwide, even between Northern and Southern European countries. In the Nordic countries trust in the quality of generics is higher than that in the Southern countries, with concerns only over their taste, packaging and appearance, since this may affect patients’ understanding and acceptance. A common concern, in particular for vulnerable patient groups (eg, elderly patients with polypharmacy or with dementia), is the confusion and problem of adherence when switching to generics. However, this drawback could be easily tackled by recommending that generic manufacturers copy not only the active ingredients, but also the originators’ excipients and packaging, in order to safeguard the “placebo effect” on generic drugs too. Furthermore, to streamline communication, INN prescribing should become mandatory and physicians and pharmacists should be educated on chemical names at the start of their training.

Another issue that does not help strengthen patients’ trust in generics is the adoption of the so-called “reference pricing” (RP) scheme, whereby health authorities set a maximum price for products that have the same active ingredient and the cost of using equivalent products that exceed this RP has to be covered by the patient [8]. This scheme, originally adopted in Germany, has been taken in many continental European countries with little resistance from the pharmaceutical industry, allowing companies some freedom in pricing their competing off-patent products. However, RP may raise concern among the general population on the real equivalence of drugs in the long term, which is often affected by the interests of physicians and pharmacists as previously discussed. The underlying and emerging message that cheaper generics are like “hard discount” low-quality products in mass markets compared with originators and so patients should be willing to pay more for “brand” products is simply false: generic drugs are equivalent to originators, so a price difference can hardly be justified on reimbursed drugs.

To conclude, although off-patent drugs have been in use for many years by definition and both their efficacy and adverse events are well known, their generic versions are still often debated, with their safety and efficacy compared with their originators continually questioned. This is hard to accept, particularly in developed countries that for decades have had clear rules in place on the characteristics of generics. Promoting prescription by INN and increasing downward pressure on prices of off-patent drugs are arguably useful tools to save on money that can be used in other ways, eg, for financing innovative drugs. Particularly in this period of financial crisis that hits both developed and developing countries, health authorities should use all means possible to constrain their budgets and generics are of utmost importance in trying to keep pharmaceutical expenditure sustainable.

## References

[1] Garattini L, Tediosi F (2000). A comparative analysis of generics markets in five European countries. Health Policy..

[2] Alfonso-Cristancho, et al. Definition and classification of generic drugs across the world. Appl Health Econ Health Policy. 2015. doi:10.1007/s40258-014-0146-1. 10.1007/s40258-014-0146-1 PMC4519628 26091708

[3] Hu, et al. A case study of pharmaceutical pricing in China: setting the price for off-patent originators. Appl Health Econ Health Policy. 2015. doi:10.1007/s40258-014-0150-5. 10.1007/s40258-014-0150-5 PMC4519586 26091710

[4] Schall, et al. The impact of generic substitution on health and economic outcomes: a review. Appl Health Econ Health Policy. 2015. doi:10.1007/s40258-014-0147-0. 10.1007/s40258-014-0147-0 PMC4519629 26091709

[5] IMS Health. Generic medicines: essential contributors to the long-term health of society. http://www.imshealth.com/imshealth/Global/Content/Document/Market_Measurement_TL/Generic_Medicines_GA.pdf. Accessed 24 Apr 2014.

[6] Hassali MA, Wong ZY, Alrasheedy AA (2014). Perspectives of physicians practicing in low and middle income countries towards generic medicines: a narrative review. Health Policy..

[7] Toverud, et al. A review of physicians’ and pharmacists’ perspectives on generic drugs and generic substitution: what are the global challenges? Appl Health Econ Health Policy. 2015. doi:10.1007/s40258-014-0145-2. 10.1007/s40258-014-0145-2 PMC4519583 25963230

[8] Giuliani G, Selke G, Garattini L (1998). The German experience in reference pricing. Health Policy..

